# Targeting Inflammatory Processes Mediated by TRPVI and TNF-α for Treating Noise-Induced Hearing Loss

**DOI:** 10.3389/fncel.2019.00444

**Published:** 2019-10-03

**Authors:** Asmita Dhukhwa, Puspanjali Bhatta, Sandeep Sheth, Krishi Korrapati, Coral Tieu, Chaitanya Mamillapalli, Vickram Ramkumar, Debashree Mukherjea

**Affiliations:** ^1^Department of Pharmacology, Southern Illinois University School of Medicine, Springfield, IL, United States; ^2^Department of Pharmaceutical Sciences, College of Pharmacy, Larkin University, Miami, FL, United States; ^3^Department of Otolaryngology, Southern Illinois University School of Medicine, Springfield, IL, United States; ^4^Department of Internal Medicine, Southern Illinois University School of Medicine, Springfield, IL, United States

**Keywords:** noise exposure, inflammation, TRPV1, TNF-α, capsaicin, Etanercept, cochlea, hearing loss

## Abstract

Noise trauma is the most common cause of hearing loss in adults. There are no known FDA approved drugs for prevention or rescue of noise-induced hearing loss (NIHL). In this study, we provide evidence that implicates stress signaling molecules (TRPV1, NOX3, and TNF-α) in NIHL. Furthermore, we provide evidence that inhibiting any one of these moieties can prevent and treat NIHL when administered within a window period. Hearing loss induced by loud noise is associated with the generation of reactive oxygen species (ROS), increased calcium (Ca^2+^) in the endolymph and hair cells, and increased inflammation in the cochlea. Increased (Ca^2+^) and ROS activity persists for several days after traumatic noise exposure (NE). Chronic increases in (Ca^2+^) and ROS have been shown to increase inflammation and apoptosis in various tissue. However, the precise role of Ca^2+^ up-regulation and the resulting inflammation causing a positive feedback loop in the noise-exposed cochlea to generate sustained toxic amounts of Ca^2+^ are unknown. Here we show cochlear TRPV1 dysregulation is a key step in NIHL, and that inflammatory TNF-α cytokine-mediated potentiation of TRPV1 induced Ca^2+^ entry is an essential mechanism of NIHL. In the Wistar rat model, noise produces an acute (within 48 h) and a chronic (within 21 days) increase in cochlear gene expression of TRPV1, NADPH oxidase 3 (NOX3) and pro-inflammatory mediators such as tumor necrosis factor-α (TNF-α) and cyclooxygenase-2 (COX2). Additionally, we also show that H_2_O_2_ (100 μM) produces a robust increase in Ca^2+^ entry in cell cultures which is enhanced by TNF-α via the TRPV1 channel and which involves ERK1/2 phosphorylation. Mitigation of NIHL could be achieved by using capsaicin (TRPV1 agonist that rapidly desensitizes TRPV1. This mechanism is used in the treatment of pain in diabetic peripheral neuropathy) pretreatment or by inhibition of TNF-α with Etanercept (ETA), administered up to 7 days prior to NE or within 24 h of noise. Our results demonstrate the importance of the synergistic interaction between TNF-α and TRPV1 in the cochlea and suggest that these are important therapeutic targets for treating NIHL.

## Introduction

Noise exposure is the most common cause of hearing loss in adults globally. It is associated with the generation of ROS in the cochlea by increasing its metabolic activity. NE decreases cochlear blood flow (CBF) (Thorne and Nuttall, 1987) and decreases cochlear oxygen tension (Hawkins, 1971). This stresses the highly metabolically active cochlea leading to increased ROS production in the cochlea (Yamane et al., 1995a; Ohlemiller et al., 1999; Yamashita et al., 2004). Yamane et al. (1995b) found that noise trauma results in an increase in superoxide anion at the luminal surface of marginal cells in the stria vascularis (SV). A secondary peak of superoxide radicals occurs hours later as a result of cochlear reperfusion and persists 7–10 days post NE. Subsequent studies confirmed that ROS increases in the cochlea following noise trauma (Henderson et al., 1999; Ohlemiller et al., 1999) and persists for several days after NE (Hu et al., 2002; Yamashita et al., 2005). This eventually leads to hair cell death that continues for days after NE (Bohne, 1976). Latest studies by Haryuna et al. (2016) report cochlear levels of H_2_O_2_ 14 days post NE (69 ± 32.99 pmol/ml) being significantly higher, compared to H_2_O_2_ levels in control unexposed rat cochlea (7.08 ± 0.96 pmol/ml), thus confirming earlier studies.

The generation of ROS by noise trauma is considered to be a critical event which initiates damage to the outer hair cells (OHCs), SV and spiral ganglion cells (SGCs), leading to hearing loss (Bohne et al., 2007; Murai et al., 2008). However, the mechanisms mediating the toxicity of ROS generation are less clearly defined. The primary source of ROS is a cochlear-specific NADPH oxidase isoform, NOX3 (Banfi et al., 2004). We previously reported that one source of ROS generation in the cochlea triggered by noise is the NADPH oxidase enzyme (Ramkumar et al., 2004). Oxidative stress in the cochlea induces the expression of NOX3 along with the non-specific cation channel, TRPV1. We have previously shown that NOX3 up-regulates TRPV1, leading to enhanced Ca^2+^ influx and inflammation in the cochlear tissues (Mukherjea et al., 2011) and that TRPV1 activation can generate ROS via NOX3 by increasing intracellular Ca^2+^ release, thus forming a feedback loop. TRPV1 has been shown to be increased in a noise-induced tinnitus model (Bauer et al., 2007). However, noise-induced increase in TRPV1 causing sustained increased calcium (Ca^2+^) in the cochlea has not been shown.

Excessive NE has been associated with increased Ca^2+^ in the endolymph and hair cells (Ikeda and Morizono, 1988; Fridberger et al., 1998; Chan and Rouse, 2016). Increased Ca^2+^ and ROS activity persisting for several days after traumatic NE are well documented (Li et al., 1997; Hu et al., 2002; Yamashita et al., 2005). Interestingly, chronic increases in Ca^2+^ and ROS has been shown to increase inflammation and apoptosis in various tissues (Nicotera and Orrenius, 1992; Orrenius et al., 1992a, b; Vass et al., 1995; Sinha et al., 2013). However, the precise role of Ca^2+^ in mediating persistent cochlear inflammation and hearing loss is not clear.

The role of inflammation in the development of hearing loss was suggested by a study showing that the administration of a corticosteroid, an anti-inflammatory agent, protected against sensorineural hearing loss (Kanzaki and Ouchi, 1981). It has been demonstrated that the cochlea itself produced these pro-inflammatory cytokines which could increase migration of inflammatory cells from the circulation to the cochlea (Hirose et al., 2005; Kim H.J. et al., 2008). ROS also activates a series of downstream events, including the activation of mitogen-activated protein kinase (MAPK), such as JNK, leading to the formation of pro-inflammatory cytokines in the cochlea and eventual apoptosis of OHCs (So et al., 2007; Bas et al., 2009). Our interest in TRPV1 was based on our early studies with freshly isolated DRG neurons. In these studies, NGF increased the expression of TRPV1 in DRG neurons by increasing ROS generation (Puntambekar et al., 2005). Increases in TRPV1 activity has been linked to inflammation in several pathological conditions (Basu and Srivastava, 2005; Razavi et al., 2006; Bertin et al., 2014; Brito et al., 2014; Liu et al., 2014) and knockdown of TRPV1 reduces cisplatin-mediated inflammation and ototoxicity (Mukherjea et al., 2008). We have also shown the synergy between TRPV1 and NOX3 in the cochlea (Mukherjea et al., 2011). Trans-tympanic (TT) administration of capsaicin (TRPV1 agonist) in the rat cochlea mediates an acute and transient increase in inflammatory markers such as TNF-α, COX2, and iNOS at 24 h, which returned to baseline values by 72 h and did not involve loss of OHC’s or apoptotic molecules (Mukherjea et al., 2011). This acute TRPV1 activation and subsequent desensitization were accompanied with a temporary threshold shift at 24 h which returned to baseline values within 72 h. Thus transient inflammation of the cochlea was associated with transient hearing loss. These findings suggest a coordinated action of TRPV1, NOX3, TNF-α and other inflammatory cytokines in mediating hearing loss, and thus are potential targets for otoprotective therapies.

Noise exposure increases the production of pro-inflammatory cytokines such as TNF-α, IL-1β, and IL6 in the cochleae (Fujioka et al., 2006; Bas et al., 2009). TNF-α, the master regulator of inflammatory cytokine cascade, is produced by spiral ligament (SL) fibrocytes, OHCs and supporting cells in the organ of Corti after NE (Zou et al., 2005; Fujioka et al., 2006). Noise exposed cochlear level of TNF-α cytokine (191.07 pg/mg) has been reported to be significantly higher when compared to untreated control levels (58.28 pg/mg) in the rat cochlea by Arslan et al. (2017). Additionally, infusion of TNF-α into the guinea pig cochlea leads to the infiltration of inflammatory cells, such as leukocytes, causing cochlear damage (Keithley et al., 2008). Organ of Corti explants exposed to TNF-α demonstrated apoptosis associated with increased expression of phosphorylated c-Jun N terminal kinase, cleaved caspase-3, apoptosis-inducing factor cluster formation, and translocation of the apoptotic marker endonuclease G to the nuclei of hair cells (Infante et al., 2012). Interestingly, the auditory protection afforded by certain antioxidants like melatonin have been linked to regulation of TNF-α in rats exposed to traumatic noise (Bas et al., 2009). Thus, homeostatic regulation of TNF-α in the cochlea is essential for normal physiological hearing.

In the present study, we show that NE initiates a cascade of events involving both acute and chronic induction of TRPV1, NOX3, and TNF-α, leading to permanent hearing loss. We show that the induction of both TRPV1 and TNF-α and the resulting synergy between these two molecules could promote intracellular Ca^2+^ overload, thus predisposing the cochlea to permanent hearing loss. In this study, we show that desensitization of TRPV1 (capsaicin) or inhibition of TNF-α (Etanercept, an FDA approved inhibitor of TNF-α) protected against NIHL in a rat model. This study, for the first time, shows that inhibition of inflammation either as pre-treatment or within a window of opportunity post noise insult can protect and treat NIHL.

## Materials and Methods

### Drugs, Reagents and Antibodies

H_2_O_2_, capsaicin, protease inhibitor, and phosphatase inhibitor cocktails 2 and 3 were purchased from Sigma-Aldrich (St. Louis, MO, United States). TNF-α cytokine was purchased from Sigma-Aldrich (St. Louis, MO, United States). Etanercept or Enbrel was purchased from Walgreens pharmacy for animal use. Various primary antibodies used were as follows: p-ERK1/2, ERK1/2, NOX3, β-actin from Santa Cruz Biotechnology (Dallas, TX, United States); TNF-α, Cox2 and iNOS from Cell Signaling Technology (Danvers, MA, United States); Alexa Fluor 488 Phalloidin from Thermo Fisher Scientific (Waltham, MA, United States). Secondary antibodies used were as follows: donkey anti-rabbit IRDye 680RD, goat anti-mouse IRDye 800RD (no. 926-32214) from LI-COR Biosciences (Lincoln, NE, United States); Alexa Fluor 488 goat anti-rabbit (no. A11008) and RNA Later were purchased from Thermo Fisher Scientific (Berkeley, MO, United States); Alexa Fluor 647 goat anti-mouse IgG1 (no. A-21240) from Molecular Probes (Eugene, OR, United States). Apoptosis was measured using ApopTag^®^ Red *In Situ* Apoptosis Detection Kit (Millipore Sigma, United States). Prolong gold anti-fade (Invitrogen) was used to mount immunohistochemistry samples for imaging.

#### Cell Culture

Immortalized mouse organ of Corti cells, UB/OC-1, were kindly provided by Dr. Matthew Holley (The University of Sheffield, United Kingdom). These cells were cultured in RPMI-1640 media (HyClone) supplemented with 10% FetalClone^®^ II serum (HyClone). UB/OC-1 cells were kept at 33°C in a humidified incubator with 10% CO2. Cells were cultured thrice a week for passaging and the sub-confluent monolayer of cells was used for experiments. HEK-VR1 (HEK cells stably transfected with TRPV1 also known as VR1) cells (Puntambekar et al., 2005) were grown in growth medium consisted of 90% DMEM, 10% fetal bovine serum (Invitrogen, Grand Island, NY, United States), and 0.2 mg/ml G418 sulfate (Calbiochem, La Jolla, CA, United States) for selection of resistance. Cells were cultured at 37°C, in the presence of 5% CO2 and 95% ambient air.

#### Calcium Assay

UB/OC-1 or HEK-VR1 cells were grown on glass coverslips to detect Ca^2+^ entry induced by TRPV1 activation. Five μm Fluo-4 AM was added to the cells for 20 min, followed by 3 rinses in the physiological buffer (HEPES:10 mM, NaCl: 130 mM, KCl 4 mM, glucose 4 mM, pH 7.3). Timed baseline fluorescent imaging was performed in calcium buffer by confocal microscopy using an argon laser at 488 nm. Live fluorescent images were recorded every 3 s for 10 min. Baseline (F0) fluorescent images were collected for the first 10 scans and then the drug was added at 30 s to obtain the relative F1 values. Data were analyzed using the Leica software as the ratio of fluorescence/baseline fluorescence and reported as the percentage of relative fluorescence compared to the baseline.

#### Apoptosis Assay

Apoptosis was detected by TUNEL assay using ApopTag^®^ Red *In Situ* Apoptosis Detection Kit. Briefly, UB/OC-1 cells were fixed with 4% paraformaldehyde, for 10 min and washed thrice with PBS. Cells were then permeabilized using proteinase K (1:100 dilution) (provided in the kit) for 20 min at room temperature. At the end of incubation, the coverslips were rinsed with 1 × Tris-buffered saline (TBS) and incubated with 1 × terminal deoxynucleotidyl transferase (TdT) equilibration buffer for 30 min. TdT labeling reaction mixture (60 μl/coverslip) was added and incubated for 1 h at 37°C. Next, the cover-slips were rinsed twice with 1 × TBS, stained with DAPI and mounted using Prolong gold anti-fade (Invitrogen) mounting medium. Excess mounting media was wiped off and the edges were sealed using nail polish. Slides were then imaged using a Leica confocal microscope.

#### Western Blotting

UB/OC-1 cells were homogenized in ice-cold lysis buffer (50 mM Tris-HCL, 10 mM Magnesium chloride and 1 mM EDTA) in the presence of protease inhibitor and phosphatase inhibitor cocktail 2 and 3. The whole-cell lysates were then used for Western blotting. After transfer to nitrocellulose membranes, blots were probed with different primary antibodies, followed by a fluorescent-tagged secondary antibody, and visualized by imaging using LICOR odyssey image. Licor Odyssey software was used to analyze the bands. The individual bands were normalized with total proteins for ERK1/2 and these were then further normalized as % of controls to 100%.

#### Animal Procedures and Sample Collection

Male Wistar rats (175–200 gm) were used for this study under an animal care protocol approved by the Laboratory and Animal Care and Use Committee (SIU School of Medicine). Pretreatment ABRs were performed. Animals were then treated according to the experimental paradigm.

Oral capsaicin (20 mg/kg) as a preventive strategy: was delivered starting 24 h prior to NE, on the day of NE, and 24 h post NE (3 consecutive treatments), and post-treatment ABR’s were collected 21 days post NE.

ETA as Prevention strategy: consisted of a single administration of ETA either trans-tympanicaly (TT) (5 μg/μl) or s.c (3 mg/kg) or PBS (TT-50 μl or s.c ∼100–200 μl) either 3 or 7 days prior to NE.

ETA as Treatment: consisted of NE followed by a single ETA treatment either TT or SC administration at either 2 h or 24 h post noise injury. Post-treatment ABR thresholds were determined at 21 days post NE and cochleae were excised for morphological, molecular and biochemical studies. Cochleae used for RNA preparations were flushed immediately with RNA Later solution and stored in RNA Later for 24 h at 4°C. Those used for immunohistochemical studies were perfused and fixed with 4% paraformaldehyde, while those used for SEM were perfused and fixed with 2.5% glutaraldehyde. Cochleae used for whole mounts were decalcified for 14–20 days in 100 mM EDTA at room temperature.

#### Noise Exposure (NE)

Male Wistar rats were exposed to octave band noise at 122 dB centered at 16 kHz for 1 h under isofluorane anesthesia, with the 3-inch silicon tubes attached to the high-frequency transducer resting in the external auditory canal. This exposure typically results in a 30–50 dB temporary threshold shift (measured as an immediate pre-to-post NE shift in ABR thresholds) across all frequencies; with slightly more elevation at those frequencies around 16 kHz and 20–40 dB permanent threshold shift measured at 21 days post NE at all frequencies. Acoustic stimuli are calibrated using a cloth model rat and a Bruel & Kjaer Pulse System with a 1/2 inch free-field microphone (B&K model 4191). Baseline noise levels in the test chamber (with background test noise turned off) are typically measured below 20 dB SPL in the 4–40 kHz range.

#### Intra-Tympanic Injections

Male rats were anesthetized with ketamine/xylazine. A 30 gauge insulin needle (5/8 inch in length) was used to puncture the tympanic membrane in the anteroinferior region. A volume of 30 μl of saline or ETA (5 μg/μl) was injected through the tympanic membrane. Rats remained undisturbed for 15 min and the procedure was repeated in the other ear (Sheehan et al., 2018).

#### Auditory Brainstem Evoked Responses (ABRs)

Pretreatment ABR thresholds were determined using the high-frequency Intelligent Hearing Systems (IHS) on naive rats prior to any treatment or NE for each ear. Animals were tested with a stimulus intensity series that was initiated at 90 dB SPL and reached a minimum of 10 dB SPL. The stimulus intensity levels were decreased in 10 dB increments, and the evoked ABR waveforms were observed on a video monitor. Threshold was analyzed by readers blinded to condition and defined as the lowest intensity capable of eliciting a replicable, visually detectable response at the ABR waveforms II and III. The auditory stimuli included tone bursts at 8, 16, and 32 kHz with a 10 ms plateau and a 1 ms rise/fall time presented at a rate of 5/s. The threshold was defined as the lowest intensity capable of evoking a reproducible, visually detectable response with two distinct waveforms and minimum amplitude of 0.5 μV.

#### Scanning Electron Microscopy (SEM)

Cochleae were carefully dissected out and sample preparation was performed as stated in Huang et al. (2007). Briefly, the round and oval windows and the apex of the cochlea were perforated with a small pick. The perilymphatic space was perfused with 2.5% glutaraldehyde in 0.1M sodium cacodylate (Cac) buffer pH 7.2, by placing a 1-mL syringe with a modified butterfly catheter over the apex. Each specimen was then placed in the glutaraldehyde solution overnight and refrigerated. Cochlear surface and perilymphatic space were rinsed three times with Cac buffer the next day. The specimen was then perfused with 1% osmium tetroxide and placed on a tissue rotator for 15 min. The sample was then rinsed in 0.1M of Cac buffer three times. Under the dissecting microscope, the bony capsule of the cochlea was carefully removed, and the lateral wall was cut away to reveal the organ of Corti over the middle turn, basal turn and hook regions. The tissue was serially dehydrated in 2 × 50%, 70%, 85%, 95%, and 3 × 100% ethyl alcohol. Each specimen was critical point dried using hexamethyldisilazane (HMDS) and placed on a stub for sputter coating with approximately 10 nm of gold/palladium alloy. The tissue was then viewed, and photographs were taken with a Hitachi S-3000N scanning electron microscope.

For each region of the cochlea, at least two representative samples of 33 OHCs (or 11 OHCs per row) was examined. The number of missing OHCs within each sample was then counted. These SEM techniques allow for a descriptive analysis of cellular scarring and stereocilia bundles. The results are presented as the percent hair cell damage per cochlear turn (Kamimura et al., 1999). OHC data were collected and counted by an experimenter blind to the treatment group.

#### Immunohistochemistry (IHC)

Cochleae used for whole mounts or mid modiolar sections were decalcified for 14–20 days in 100 mM EDTA at room temperature and either microdissected or mounted into OCT and cryosectioned. For IHC processing, primary antibody (1:100 titer) and secondary fluorescent-labeled antibodies (1:200 titer) were used. Slides were imaged by Leica confocal microscope (Leica America).

#### RNA Isolation

Cochleae were pared down to the bone to remove all extraneous tissue, crushed in liquid nitrogen followed by extraction in 500 μl of TRI reagent. 0.1 ml of chloroform was added, and the tube was shaken vigorously for 15 s and centrifuged at 12,000 × *g* for 15 min. RNA was extracted by washing the pellet with 0.5 ml ice-cold isopropanol followed by cold 75% treated ethanol. The ethanol was removed and the tube was air-dried briefly. The RNA pellet was resuspended in nuclease-free water and RNA levels were determined using optical density readings corresponding to wavelengths of 260, 280, and 230 nm using a spectrophotometer (Eppendorf BioPhotometer, Hamburg, Germany).

#### Real-Time RT-PCR

Five hundred nanogram of total RNA was converted to cDNA using the iScript cDNA Synthesis Kit (Bio-Rad, Hercules, CA, United States). The reaction mixture was set up as follows: 500 ng of total RNA, 4 μl of iScript reaction mix, 1 μl of iScript reverse transcriptase, nuclease-free water to bring the total volume to 20 μl. The reaction mix was incubated at 25°C for 5 min, 42°C for 30 min and 85°C for 5 min. This cDNA reaction mix was used for real-time PCR, as described previously (Mukherjea et al., 2011). Gene-specific primer pairs were used for the various reactions and mRNA expression levels were normalized to the levels of GAPDH. The primer sets were purchased from Sigma Genosys (St. Louis, MO, United States), and were as follows:

Rodent-GAPDH (sense): 5′-ATGGTGAAGGTCGGTGTG AAC-3′
  (antisense): 5′-TGTAGTTGAGGTCAATGAAGG-3′
Rodent TRPV1 (sense): 5′-CAAGGCTGTCTTCATCA TC-3′
  (antisense): 5′-AGTCCAGTTTACCTCGTCCA-3′
Rodent NOX3 (sense): 5′-GTGAACAAGGGAAGGCT CAT-3′
  (antisense): 5′-GACCCACAGAAGAACACGC-3′
Rodent-TNF-α (sense): 5′-CAGACCCTCACACTCAGA TCA-3′
  (antisense): 5′-TGAAGAGAACCTGGGAGTAGA-3′
Rodent-COX2 (sense): 5′-TGATCGAAGACTACGTGCA AC-3′
  (antisense): 5′-GTACTCCTGGTCTTCAATGTT-3′
Rodent iNOS (sense): 5′-AAGTACGAGTGGTTCCA GGA-3′
  (antisense): 5′-GCACAGCTGCATTGATCTCG-3′

### Statistical Analysis

Statistical significance differences among groups were performed using either student’s *t*-Test or ANOVA, followed by Tukey’s *post hoc* test. *p* < 0.05 was considered to be statistically significant.

## Results

### Noise Exposure Increases Cochlear Inflammatory Genes

**FIGURE 1:**
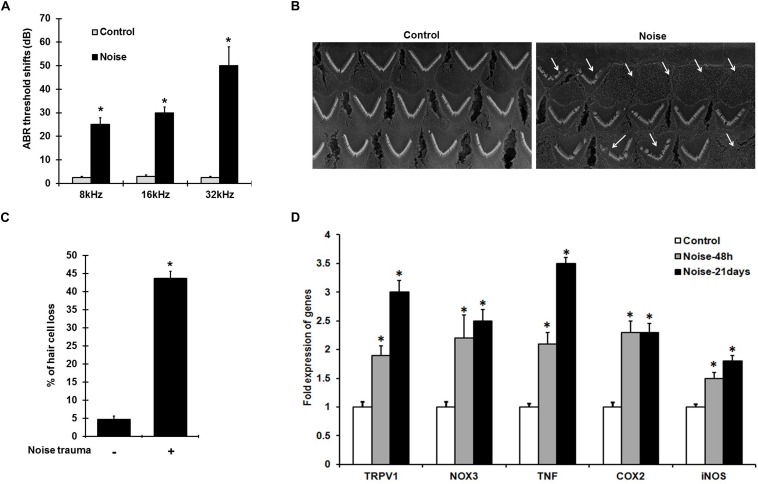
NIHL is associated with persistent increases in gene expression of pro-inflammatory mediators. **(A)** Pre-treatment ABR’s were performed and rats were noise exposed. Post treatment ABR thresholds shifts, recorded 21 days after noise exposure were 25 ± 4, 30 ± 3, and 52 ± 7 dB at 8, 16, and 32 kHz respectively. **(B)** SEM of the basal turn of cochlea showing damage/loss OHCs (see arrows) after 21 days. **(C)** Graphical representation showing damage/loss of 43 ± 4% of OHCs (*n* = 6), compared to controls. **(D)** Noise increased the expression of stress-responsive and inflammatory genes at 48 h and 21 days post noise exposure. Significant increases in all the genes (normalized to *GAPDH*) were observed compared to control cochleae. Asterisk (^∗^) denotes statistically significant difference between noise-exposed and control cochlea. Statistical significance was analyzed using student’s *t*-Test or ANOVA and Tukey’s *post hoc* analysis.

Noise exposure induces ABR threshold shifts of 25–50 ± 8 dB in rats at 8, 16, and 32 kHz tested (Figure 1A), and is associated with increased cochlear TRPV1 and TNF-α expression. Scanning electron microscopic (SEM) images of the organ of Corti show ∼40–50% damage/loss of OHCs (Figures 1B,C) in the noise-exposed animals. The expression of cochlear stress and inflammatory genes measured by qPCR at 48 h and 21 days following NE were elevated in a time-dependent manner. Interestingly, the increases for TRPV1 and TNF-α were significantly greater at 21 days than at 48 h. TRPV1 expression was increased to 2.0 ± 0.2 fold at 48 h and further increased to 3 ± 0.2 fold by 21 days while TNF-α expression increased from 2.1 ± 0.2 to 3.5 ± 0.2 fold over the same time period. NOX3, iNOS, and COX2 expression increased significantly at 48 h to 2.2 ± 0.4, 1.5 ± 0.2, and 2.3 ± 0.2 fold, respectively, with no significant changes at 21 days post-NE (Figure 1D). These findings demonstrate the induction of TRPV1, TNF-α, and NOX3 as early and persistent markers of NIHL.

### ROS Increases TRPV1-Dependent Ca^2+^ Entry Which Is Potentiated by TNF-α

**FIGURE 2:**
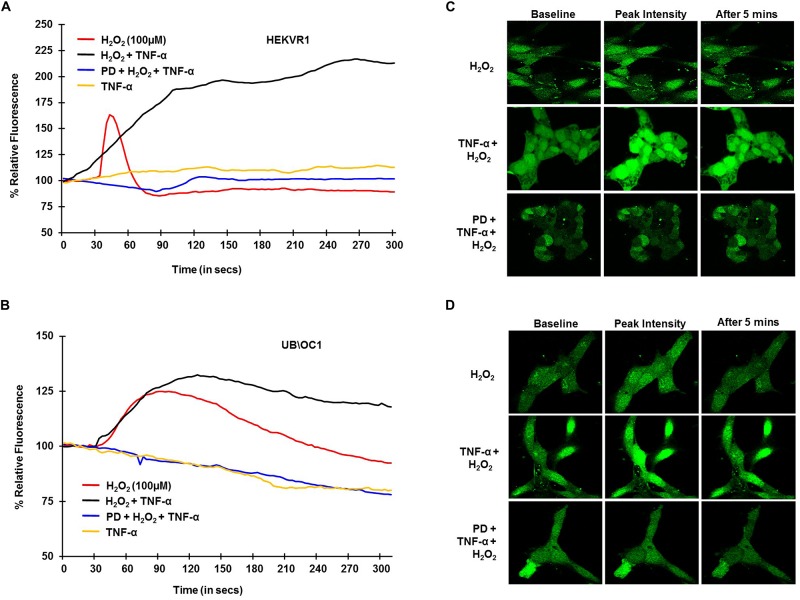
TRPV1 mediated Ca^2+^ release by ROS is potentiated by TNF-α and requires ERK activation. HEKVR1 or UB/OC-1 cells were loaded with Fluo-4AM dye and baseline fluorescence was recorded every 3 s by confocal microscopy. Graphical representations of % relative fluorescence was calculated using F/F0 ratios. For TNF-α studies, 0.1 ng/ml of this cytokine was added, followed by addition of 100 μM H_2_O_2_ after 60 s and fluorescence recorded for 5 min. **(A,B)** H_2_O_2_ elicits a rapid robust Ca^2+^ response in HEKVR1 **(A)** as well as UB/OC-1 cells **(B)** and this was potentiated by pretreatment with TNF-α. Pretreatment with ERK inhibitor-PD98059 (10 μM) for 30 min inhibits ROS mediated Ca^2+^ response completely, confirming the role of *ERK activation* in both cell lines. **(C,D)** Are representative images of the Ca^2+^ response of the two cell lines (**C**: HEKVR1 cells, **D**: UB/OC-1 cells) used in the study.

To establish the role of TRPV1 channel in NIHL in, *in vitro* studies and examine whether TNF-α potentiates TRPV1-induced Ca^2+^ influx, H_2_O_2_ was used to mimic ROS produced following NE. HEKVR1 and immortalized organ of Corti (UB/OC-1) cells were used. The cells were loaded with Ca^2+^ dye - Fluo-4AM washed and imaged every 3 s for 300 s. Basal fluorescence was captured for 10 scans and 100μM H_2_O_2_ was added at 30 s and the resulting fluorescence captured. H_2_O_2_ treatment elicited a rapid robust Ca^2+^ response within 10 s which returned to baseline by 60 s. Similar robust Ca^2+^ response was seen in UB/OC-1 cells starting at 30 s that returned to baseline by 3 min. The differences in temporal profile are due to fewer TRPV1 channels in UB/OC-1 cells (data not shown) compared to HEKVR1 cells. Pretreatment with TNF-α (0.1 μg/ml) for 10 min prior to treatment with H_2_O_2_ resulted in a prolonged and sustained Ca^2+^ fluorescence in both HEKVR1 and UB/OC-1 cells which is observed until 5 min (Figures 2A,B). Pictorial representation of baseline, maximal and fluorescence after 5 min is shown in HEKVR1 cells (Figure 2C) and in UB/OC-1 cells (Figure 2D). Since extracellular signal-regulated kinase (ERK) activation contributes to the pathology of NIHL (Meltser et al., 2010; Maeda et al., 2013), we tested the involvement of this kinase in TRPV1-mediated Ca^2+^ influx by pre-treating the ERK inhibitor, PD98059. Our data indicate that TRPV1-mediated Ca^2+^ entry is ERK1/2 dependent, as pretreatment of the cells with 10 μM PD98059 blunted the Ca^2+^ release in both HEKVR1 as well as UB/OC-1 cells (Figures 2A,B).

**FIGURE 3:**
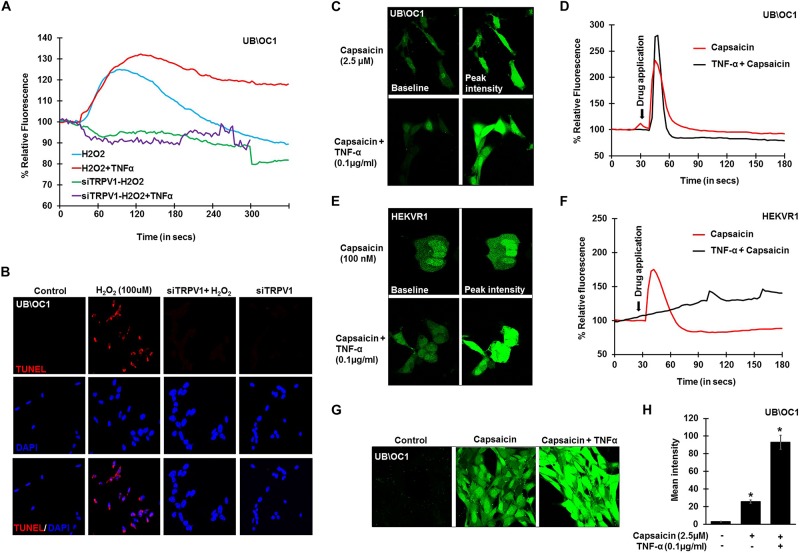
TNF-α potentiates direct TRPV1 activation. UB/OC-1 or HEKVR1 cells were plated on cover slips for these experiments. **(A)** UB/OC-1 cells were treated with siTRPV1 (10 nM) or siScramble (5 nM) for 48 h, loaded with Fluo-4AM dye and treated with 100 μM H_2_O_2_ and imaged every 3 s for 5 min. siTRPV1 treatment completely abolishes H_2_O_2_ mediated Ca^2+^ release, while siScramble had no effect on H_2_O_2_ mediated Ca^2+^ release. Pre-treatment with TNF-α (0.1 ng/ml) for 10 min followed by 100 μM H_2_O_2_ did not elicit any Ca^2+^ response, indicating that TRPV1 is essential in ROS mediated Ca^2+^ release. **(B)** Knockdown of TRPV1 by siRNA inhibits H_2_O_2_ mediated apoptosis in UB/OC-1 cells. UB/OC-1 cells were treated with siTRPV1 (10 nM) or siScramble (5 nM) for 48 h prior to H_2_O_2_ (100 μM) for another 48 h. The reaction was then stopped and cells fixed with freshly prepared 4% paraformaldehyde and apoptosis was detected by TUNEL staining. H_2_O_2_ treatment for 48 h shows 74 ± 7% cell death which was abolished by knockdown of TRPV1. **(C–F)** UB/OC-1 or HEKVR1 cells loaded with Fluo-4AM were either pre-treated with TNF-α 0.1 ng/ml or PBS for 10 min, and baseline scanned for fluorescence for 10 scans, treated with TRPV1 direct agonist capsaicin (2.5 μM for UB/OC-1 or 100 nM for HEKVR1) and imaged every 3 s for 3 min. Capsaicin elicits a sharp and robust Ca^2+^response which was further potentiated by addition of TNF-α cytokine. **(C)** Pictorial representation of UB/OC-1 cells eliciting a sharp and robust response to capsaicin (2.5 μM) which was further induced by TNF-α pretreatment. **(D)** Graphical representation of Ca^2+^response in UB/OC-1 cells. **(E)** Pictorial representation of HEKVR1 cells eliciting a sharp and robust response to capsaicin (100 nM). Interestingly, TNF-α pretreatment, elicits a persistant Ca^2+^response in HEKVR1 cells. **(F)** Graphical representation of Ca^2+^response in HEKVR1 cells. **(G,H)** Pictorial and graphical representation of UB/OC-1 cells showing a tremendous increase in Ca^2+^ release with capsaicin treatment over basal fluorescence, which was further increased with the addition of TNF-α. Asterisk (^∗^) denotes statistically significant difference compared to untreated control.

To further confirm the involvement of TRPV1 in H_2_O_2_ mediated Ca^2+^ entry, knockdown of TRPV1 was achieved by transfecting the UB/OC-1 cells with siRNA for TRPV1 (Mukherjea et al., 2008). Knockdown of TRPV1 by siTRPV1 abolished the Ca^2+^ response in UB/OC-1 cells by H_2_O_2_ and pretreatment with TNF-α did not have any effect, suggesting that H_2_O_2_-stimulated Ca^2+^ entry was mediated via TRPV1 (Figure 3A). Furthermore, inhibition of TRPV1 by transfection of UB/OC-1 cells with siRNA inhibited apoptosis induced by 100 μM H_2_O_2_ as seen by TUNEL staining (Figure 3B). To further confirm the role of TNF-α in potentiating the Ca^2+^ response via the TRPV1 channel, UB/OC-1, and HEKVR1 cells were loaded with Fluo-4AM dye and pretreated with either PBS or TNF-α (0.1 μg/ml) for 10 min. Addition of capsaicin (2.5 μM for UB/OC-1 cells and 100 nM for HEKVR1 cells) produced a robust increase in Ca^2+^ uptake in both UB/OC-1 and HEKVR1 cells compared to capsaicin alone (Figures 3C,D for UB/OC-1 and Figures 3E,F for HEKVR1). More prolonged exposure of UB/OC-1 cells to the capsaicin + TNF-α combination for 30 min increased baseline fluorescence from 100 ± 2% to 741 ± 22% with capsaicin treatment to 3,146 ± 532% increase with capsaicin + TNF-α (Figures 3G,H). These data indicate that ROS (such as H_2_O_2_) can activate TRPV1 to increase intracellular Ca^2+^ accumulation and that this action is potentiated by TNF-α via an ERK 1/2 dependent pathway in the cochlea.

### ERK Activation Is Essential for ROS Mediated Apoptosis and in TNF-α Mediated Inflammation

**FIGURE 4:**
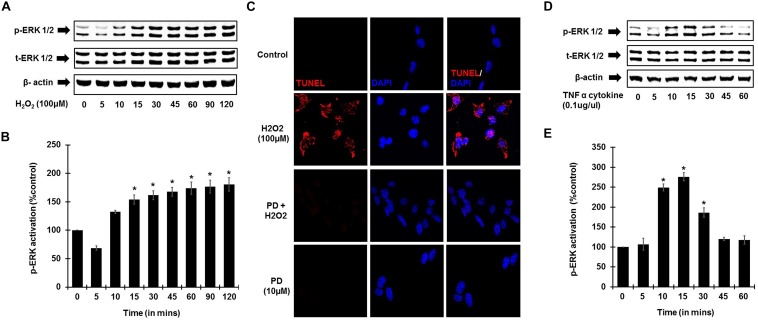
ROS and TNF-α activate ERK phosphorylation. UB/OC-1 cells were used for these assays. **(A)** ROS activates ERK phosphorylation in UB/OC-1 cells. UB/OC-1 cells were treated with 100 μM H_2_O_2_ and probed for phospho-ERK1/2 activation by western blotting and normalized to total ERK, with β-actin acting as loading control. Phospho-ERK activation was elicited in a time dependent manner, with significantly increased expression seen up to 120 min post treatment. **(B)** Graphical representation of ROS mediated ERK activation in UB/OC-1 cells. **(C)** ROS induced apoptosis is ERK dependent. UB/OC-1 cells were pre-treated with vehicle or ERK inhibitor PD98059 (10 μM) for 45 min prior to treatment with 100 μM H_2_O_2_ for 48 h. H_2_O_2_ treatment showed 80 ± 8% cell death, which was significantly decreased by pretreatment with ERK inhibitor PD98059 as observed by TUNEL staining. **(D,E)** TNF-α (0.1 μg/ml) treatment elicits ERK activation in UB/OC-1 cells. ERK activation by TNF-α (0.1 μg/ml) treatment elicited a bell shape curve with highest expression seen at 15 min post treatment in UB/OC-1 cells (*p* < 0.05, ^∗^ indicates statistically significant compared to control).

To establish the role of ROS activation of ERK phosphorylation, UB/OC-1 cells were treated with H_2_O_2_ (100 μM). Increase in ERK1/2 phosphorylation in UB/OC-1 cells treated with 100 μM H_2_O_2_ was observed in a time-dependent manner (Figures 4A,B), with significantly elevated expression seen up to 120 min post-treatment. Additionally, inhibition of ERK activation by PD98059 prior to treatment with 100 μM H_2_O_2_ showed significantly decreased cell death by TUNEL (Figure 4C). Interestingly, TNF-α (0.1 μg/ml) treatment alone significantly activates ERK 1/2 phosphorylation and elicited a bell-shaped curve with the highest expression seen at 15 min post-treatment (Figures 4D,E).

### Desensitization of TRPV1 by Capsaicin Administration Prevents NIHL

**FIGURE 5:**
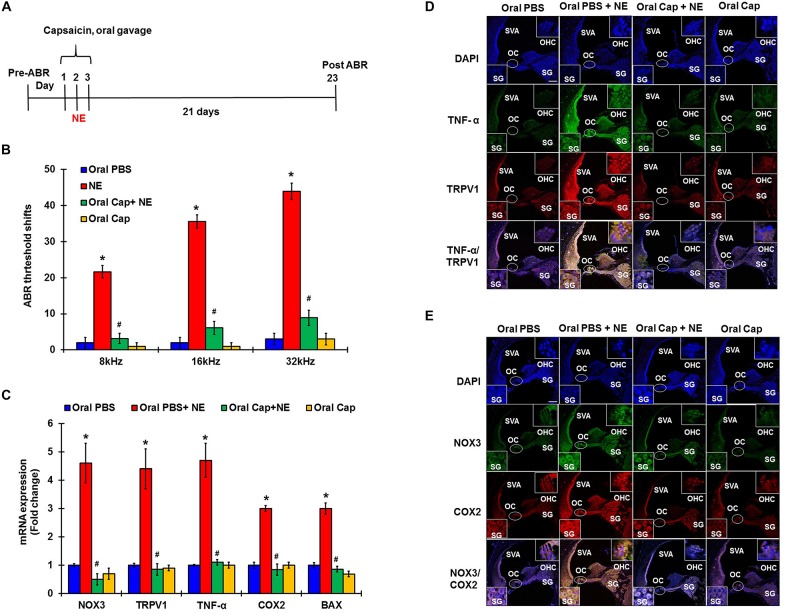
Desensitization of TRPV1 by capsaicin pretreatment abrogates NIHL. Pretreatment ABRs were performed, followed by oral capsaicin and noise exposure. Post treatment ABR threshold were recorded 21 days after noise exposure. **(A)** Schematic diagram of animal treatments. **(B)** Noise exposure showed 25–45 dB threshold shift at 8, 16, and 32 kHz, which was abrogated by oral capsaicin. Asterisk (^∗^) denotes statistically significant difference from the noise + vehicle group. Statistics were performed using ANOVA and Tukey’s *post hoc* analysis, *N* = 6. **(C)** Gene expression analyses of the various stress response, inflammatory and apoptotic markers by qPCR of the rat cochleae showed that oral capsaicin treatment inhibits the upregulation of all the stress markers by NE. Fold increases in gene expressions by NE over oral PBS treated control rat cochleae were as follows: TRPV1 (4.4 ± 0.7), TNF-α (4.7 ± 0.6), NOX3 (4.6 ± 0.7), COX2 (3 ± 0.2), Bax (3 ± 0.1), while the oral Cap + NE group showed basal levels of gene expression where TRPV1 (0.85 ± 0.2), TNF-α (0.9 ± 0.1), NOX3 (0.5 ± 0.2), COX2 (0.84 ± 0.2) and Bax (0.86 ± 0.1). Oral Capsaicin treatment alone showed basal levels of all genes namely: TRPV1 (0.9 ± 0.1), TNF-α (1 ± 0.15), NOX3 (0.7 ± 0.2), COX2 (1 ± 0.1) and Bax (0.69 ± 0.1). **(D,E)** Immunolabelling of rat cochlear mid-modiolar sections was performed 21 days post noise exposure. Noise exposure increased TRPV1, TNF-α, NOX3 and COX2 immunoreactivity, which was significantly decreased by oral capsaicin pretreatment. Scale bars represent 50 μm (^∗^ shows significant difference compared to oral PBS group, ^#^ shows significant difference compared to NE).

In these studies, pre-treatment ABRs from naïve rats were recorded and rats were then treated with daily oral PBS or capsaicin starting 24 h prior to noise, on the day of NE, and at 24 h post NE. The final ABRs were recorded 21 days post NE. Schematic representation of the treatment paradigm has been depicted in Figure 5A. The noise produced ABR threshold shifts of 21.6 ± 1.7, 35.5 ± 1.9, and 43.9 ± 2.3 dB at 8, 16, and 32 kHz, respectively. Oral capsaicin reduced threshold shifts to 3.9 ± 1.4, 6.1 ± 1.8, and 8.9 ± 2.1 dB at 8, 16, and 32 kHz respectively. Oral capsaicin administration did not show any significant ABR threshold shift compared to control (1 ± 1.1, 1 ± 1.1, 3 ± 1.6 dB threshold shift at 8, 16, and 32 kHz respectively). Statistical significance was calculated using one way ANOVA with Tukey’s *post hoc* analyses, *p* < 0.05, *N* = 6 (Figure 5B). Gene expression analyses of the various stress response, inflammatory and apoptotic markers by qPCR of the rat cochleae showed that oral capsaicin inhibits the upregulation of all the stress markers by noise (Figure 5C). Fold increases in gene expressions by noise over oral PBS treated control rat cochleae were 4.4 ± 0.7 fold for TRPV1, 4.7 ± 0.6 for TNF-α, 4.6 ± 0.7 for NOX3, 3 ± 0.2 for COX2 and 3 ± 0.1 for Bax, while the cochleae from rats administered oral capsaicin + noise group showed basal levels of gene expression and oral capsaicin treatment alone showed basal levels of all genes tested (Figure 5C). Fluorescent immunolabelling of the mid-modiolar sections of the rat cochleae with TRPV1 and TNF-α antibody shows a robust increase in fluorescence in cochleae from noise-exposed rats but not in cochleae from rats administered oral capsaicin followed by noise (Figure 5D). Additionally, immunofluorescent staining of inflammatory mediators such as NOX3 and COX2 also show a similar decrease in expression in rat cochleae pre-treated with oral capsaicin (Figure 5E). Thus, capsaicin protects against NIHL and associated cochlear inflammatory markers.

### TNF-α Inhibition Can Prevent and Treat NIHL

**FIGURE 6:**
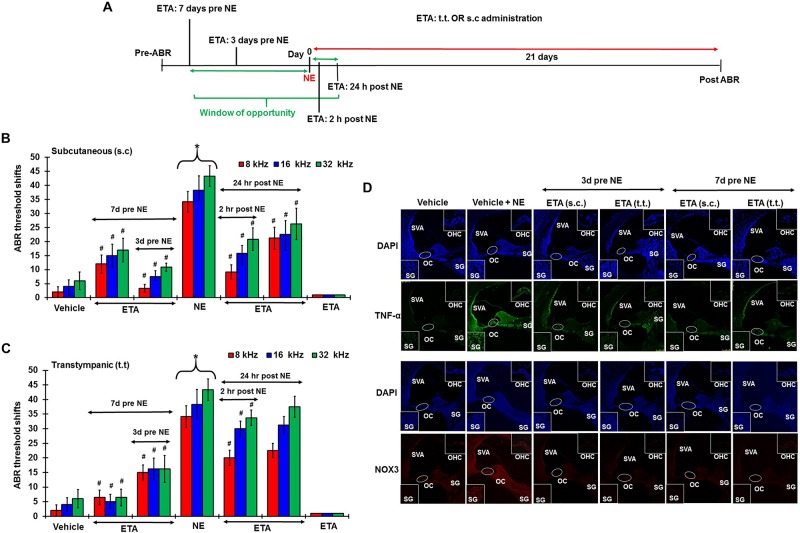
Etanercept can prevent and treat unanticipated NIHL. Pretreatment ABRs were performed in naïve Wistar rats. Treatment were performed either according to the prevention paradigm or the rescue paradigm. Post treatment ABR thresholds were recorded 21 days after noise exposure for both paradigms. ABR threshold shift was measured. Noise trauma demonstrates a 25–60 dB elevation in hearing threshold. **(A)** Schematic diagram of animal treatments. **(B,C)** Graphical representation of ABR threshold shifts measured at 21 days post NE and were grouped according to route of administration (**B**: s.c administration, and **C**: TT administration). Pretreatments with either route of administration show similar degree of prevention from NIHL when administered at either 7 or 3 days before NE. Rescue treatments indicate better protection via the sub-cutaneous route when administered within 24 h post NE. Rescue treatment with transtympanic administration of ETA at 2 h also elicits significant protection from NIHL, though the protection is not as strong as the s.c treatment administered at the same time point. Rescue treatment with TT ETA at 24 h post NE elicits ABR threshold shifts that are lower than NE alone, however, significance is only seen at 8 kHz frequency. **(D)** ETA pretreatment down regulates noise exposure induced TNF-α and NOX3 protein expression in the cochleae as seen by immunolabelling of mid-modiolar sections (^∗^ shows significant difference compared to oral PBS group, ^#^ shows significant difference compared to NE).

To determine whether inhibition of the inflammatory TNF-α signaling could ameliorate NIHL, we administered ETA by either TT or s.c injections. The drug treatment was administered either as a single preventive pretreatment given prior to noise or as a single rescue treatment administered after noise. Schematic representation of the 4 different treatment paradigms have been shown in Figure 6A. We hypothesized that sequestering TNF-α by ETA pretreatment either 3 days or 7 days prior to NE should diminish TNF-α-mediated inflammation in the cochlea. Further, in the event of unanticipated NE, rescue ETA treatment if given within a window period of either 2 or 24 h post-NE, would alleviate the TNF-α induced inflammation and ameliorate NIHL.

Single ETA administration (TT-150 μg/30 μl, or s.c: 3 mg/kg) or sterile PBS (TT-30 μl, s.c:1 ml) was performed either before or after NE according to the experimental design. Post-treatment ABR assessments were performed 21 days following NE. The cochleae were collected and processed for various biochemical and immunohistochemical assays. At least four animals were used per treatment group. Noise produced 36.25 ± 3, 45 ± 3, and 48.75 ± 2.5 dB threshold shifts, recorded at 8, 16, and 32 kHz. Gene expression analyses by real-time RT-PCR showed that noise increased the expression of cochlear TRPV1, NOX3, TNF-α, iNOS and COX2 by ∼2–5 fold. ETA was administered either 7 or 3 days prior to NE by either TT or s.c administration. The 7 days pretreatment paradigm showed significant protection from NIHL with threshold shifts measuring at s.c ETA being 12.5 ± 3.6, 15 ± 5, and 16.25 ± 5.3 dB at 8, 16, and 32 kHz, respectively. The TT ETA produced threshold shifts of 16.3 ± 3.3, 16.3 ± 3.8, and 16.3 ± 4.6 dB at 8, 16, and 32 kHz, respectively (Figures 6B,C). Immunohistochemistry of mid-modiolar sections of treated animals show decreased TNF-α and NOX3 staining by both routes of administration compared to NE group alone (Figure 6D). Similar otoprotection was achieved by ETA administered 3 days prior to NE (Figures 6B,C).

**FIGURE 7:**
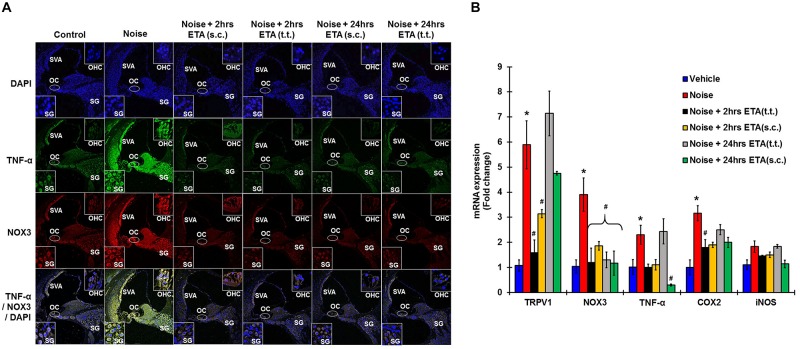
Etanercept rescue treatment down regulates noise induced inflammation in a time dependent manner. Rescue paradigm: Noise exposure (OBN 122 dB, centered at 16 kHz for 1 h) was performed, and ETA treatment was rendered at 2 or 24 h post NE (by either sc. or TT injections). Cochleae were collected 21 days after noise exposure and processed for immunohistochemistry or gene expression studies by qPCR. **(A)** Mid-modiolar sections were immunolabelled for TNF-α (green) and NOX3 (red). Noise exposure increased the expression of these proteins in spiral ligament, stria vascularis, organ of Corti and in the spiral ganglion cells. Etanercept treatment down regulates the expression of TNF-α and NOX3 in all treatment groups. **(B)** Cochlear gene expression studies by qPCR were conducted for TRPV1, NOX3, TNF-α, COX2, and iNOS. GAPDH was used as the housekeeping gene. Noise exposure increased the expression of these genes to (5.9 ± 0.96, 3.9 ± 0.66, 2.3 ± 0.38, 3.2 ± 0.3, 1.83 ± 0.23) for TRPV1, NOX3, TNF, COX2, and iNOS respectively. TT-ETA treatment when administered at 2 h post NE elicits (1.6 ± 0.4, 1.2 ± 0.57, 1 ± 0.13, 1.45 ± 0.03, 1.8 ± 0.30), s.c-ETA treatment at 2 h post NE elicits (3.13 ± 0.16, 1.9 ± 0.17, 1.1 ± 0.21, 1.5 ± 0.11, 1.9 ± 0.2), TT-ETA treatment when administered at 24 h post NE elicits (7.14 ± 0.89, 1.3 ± 0.32, 2.44 ± 0.5, 1.84 ± 0.07, 2.5 ± 0.2), while s.c-ETA treatment at 24 h post NE elicits (4.75 ± 0.07, 1.2 ± 0.48, 0.3 ± 0.04, 1.14 ± 0.15, 2 ± 0.20) for TRPV1, NOX3, TNF, COX2 and iNOS respectively. It was interesting to note that ETA treatment abolishes the TNF-α and NOX3 gene expression in all groups except for the TT-ETA at 24 h, which reflects ABR threshold shifts and the degree of protection established or lack thereof (^∗^ shows significant difference compared to oral PBS group, ^#^ shows significant difference compared to NE).

To determine whether ETA treatment could be used to alleviate or lessen the extent of NIHL due to unanticipated NE and provide a window of opportunity for treatment, we administered a single dose of ETA 2 h or 24 h after noise by either TT or s.c routes. The treatment paradigms are depicted schematically in Figure 6A. We show that ETA administered within 2 h of noise by the s.c route provides significantly better protection from NIHL compared to the TT route (s.c. ETA: 9.16 ± 2.6, 15.8 ± 2.8 and 20.8 ± 4.0 dB threshold shifts) compared to TT route of administration (20 ± 2.6, 30 ± 2.7 and 33.75 ± 2.7 dB threshold shifts) at 8, 16, and 32 kHz respectively (Figures 6B,C). Immunolabelling of the mid-modiolar sections of the rat cochleae for TNF-α and NOX3 indicate that ETA treatment decreases the expression of these proteins in the cochlea. Gene expression analyses of the stress response genes also show decreased expression of TNF-α and NOX3 compared to noise. Interestingly, TRPV1 gene expression though lower than NE are still high. Initiation of ETA 24 h post NE by the s.c route provided significantly greater protection from NIHL with threshold shifts of (s.c. ETA: 21.25 ± 3.9, 22.5 ± 4.9, and 26.25 ± 5.6 dB) compared to trans-tympanic administration (TT ETA: 22.5 ± 2.5, 31.25 ± 2.9, and 37.5 ± 3.6 dB) at 8, 16, and 32 kHz respectively (Figures 6B,C). Immunolabelling of the cochlear sections for TNF-α and NOX3 indicate decreased expression of these two proteins (Figure 7A). Gene expression studies also show decreased cochlear TNF-α and NOX3, however, the levels of TRPV1 and COX2 are not significantly different than NE (Figure 7B). We believe that the increased TRPV1 and COX2 gene expression levels signify that the cochlea is severely compromised and TNF-α sequestration at 24 h (especially for the TT administration) is too late to reverse/significantly decrease the chronic inflammatory cascade.

## Discussion

The goal of this study was to determine the salient mechanism of NE mediated NIHL and to identify tangible targets for otoprotective therapy. NE has historically been associated with increased ROS generation (Yamane et al., 1995a; Ohlemiller et al., 1999), increased Ca^2+^ in the endolymph and hair cells (Ikeda and Morizono, 1988; Fridberger et al., 1998; Chan and Rouse, 2016), and increased inflammation (Zou et al., 2005; Fujioka et al., 2006) in the cochlea in several different animal models. Increased Ca^2+^ and ROS activity have been reported to persist for several days after traumatic NE (Li et al., 1997; Hu et al., 2002; Yamashita et al., 2005). However, a direct link between ROS, Ca^2+,^ and inflammation has not yet been established. In this present study, we proposed that development of NIHL is associated with an acute as well as a chronic increase in ROS, Ca^2+,^ and inflammation and that inhibition of any one arm of this triad of events by repurposing FDA approved drugs is a viable translatable solution.

Our hypotheses were based on observation of a cascade of events occurring early after NE. NE produces an early increase in cochlear NOX3, TRPV1 and inflammatory mediators (TNF-α, iNOS, and COX2) in the rat at 48 h that persisted for at least 21 days. The expression of TRPV1 and TNF-α were significantly higher at 21 days compared to 48 h post NE and led us to speculate that these genes, along with NOX3 were an essential part of a positive feedback loop for the development of NIHL.

### Proposed Mechanism of Action

**FIGURE 8:**
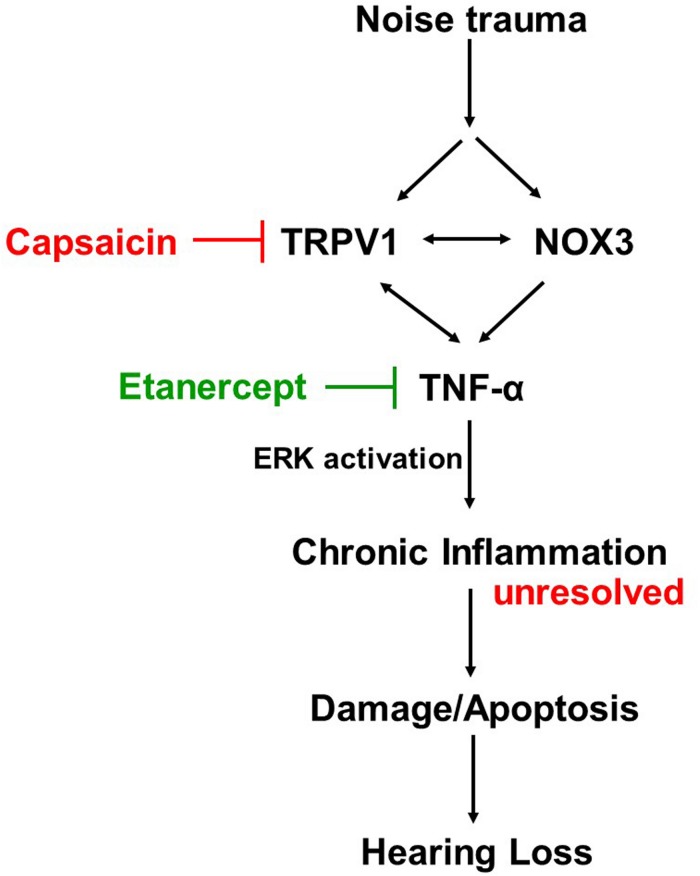
Proposed mechanism of prevention and treatment of NIHL. Noise exposure increases stress response molecules TRPV1, cochlear specific NADPH oxidase NOX3 and inflammatory mediators such as TNF-α, iNOS and COX2, acutely (within 48 h) and chronically (21 days post noise exposure). Inhibition of either TRPV1 by desensitization with oral capsaicin or inhibition of TNF-α with Etanercept (TNF-α inhibitor) show amelioration of noise induced hearing loss.

Cochlear injury induced by noise trauma activates and increases NOX3 and TRPV1 expression which contributes to increases in TNF-α production, ERK activation, and inflammation. Unresolved chronic inflammation results in damage or death of cells in the cochlea and hearing loss. Blocking any one of these moieties of the pathway will prevent and treat NIHL (Figure 8).

Chronic TRPV1 channel activation leads to chronic increases in intracellular Ca^2+^ accumulation. Thus, induction of TRPV1 by noise could lead to Ca^2+^ overload in cochlear structures expressing these channels. Interestingly, chronic increases in Ca^2+^ and ROS have been shown to increase inflammation and apoptosis in various tissues (Nicotera and Orrenius, 1992; Orrenius et al., 1992a, b; Vass et al., 1995; Sinha et al., 2013). Our studies of cisplatin-induced hearing loss have implicated TRPV1 activation in the generation of ROS via NOX3, inflammation, and apoptosis in the cochlea (Mukherjea et al., 2008; Mukherjea et al., 2011). In the present study, we have shown that noise-induced ROS mimicked by H_2_O_2_ *in vitro*, can activate TRPV1 channel and increase Ca^2+^ influx in an immortalized organ of Corti cell line UB/OC-1 as well as in HEKVR1 cells. However, when these cell lines were pretreated with TNF-α for 10 min, the addition of H_2_O_2_ elicited a strong persistent opening of the TRPV1 channel. Thus, we show that NE not only dysregulates the TRPV1 channel expression but that the accompanying inflammation as mediated by TNF-α (and possibly other cytokines) will further potentiate this action, eventually leading to cell death. The novelty of this observation is that this is the first time TNF-α mediated potentiation of TRPV1 mediated Ca^2+^ entry has been implicated in hearing loss. Similar phenomenon of TNF-α induced potentiation of Ca^2+^ current evoked by TRPV1 has been reported in rat pulmonary sensory neurons in asthma (Hu et al., 2010), in cultured trigeminal ganglions isolated from neonatal rats (Meng et al., 2016), in human synoviocytes (Kochukov et al., 2009) and in sensitization of the spinal cord in the pain model (Spicarova and Palecek, 2010).

Direct TRPV1 agonist capsaicin can activate and subsequently desensitize the TRPV1 receptor rapidly. Interestingly, our data indicate that rats pre-treated with oral capsaicin prior to noise, show complete protection from ABR threshold shifts. Protein, as well as gene expression studies of the cochleae of these animals, indicate that pretreatment with capsaicin not only abolishes the noise-induced increases in TRPV1 but also inhibits NOX3, TNF-α, COX2, and iNOS. It was interesting to note that capsaicin administered prior to noise suppresses ROS generation as well as inflammation.

Our next hypothesis was that if inflammation due to TNF-α were to be inhibited prior to NE, the degree of NIHL will be ameliorated. We further hypothesized that in the event of unanticipated NE, TNF-α sequestration within a limited time window will afford some degree of protection from NIHL. We used ETA, a fusion protein consisting of two p75 TNF receptors bound to human IgG1receptor (Mohler et al., 1993) and currently used to treat autoimmune diseases like plaque psoriasis, psoriatic arthritis, ankylosing spondylitis among others. Prophylactic treatment with ETA performed either 3 or 7 days prior to noise, significantly protected from the ABR threshold shift measured at 21 days post NE. It appears that either systemic administration by subcutaneous injections or local delivery by TT injection were equally effective at protecting from NIHL when given 7 days prior to noise. Similarly, both routes of administration are equally effective in preventing NIHL when given 3 days prior to NE. The 3 days ETA pre-treatment regimen shows stronger protection than the 7 days pre-treatment regimen. This is a very interesting observation that inhibition of inflammation either systemically or locally prior to noise would ameliorate noise-induced ABR threshold shift. This implies that when used prophylactically in an uninjured body with basal levels of inflammation, the availability of a TNF-α antagonist can reduce the local levels of TNF-α and this prevents subsequent up regulation of TRPV1, thus lessening the impact of traumatic noise on the cochlea. It also indicates that lower levels of TNF-α in systemic circulation prevent recruitment of immune-competent cells by the cochlea. Lower levels of circulating TNF-α would also modulate the expression of adhesion molecules such as ICAM-1 in the cochlea (Kim H. et al., 2008) which have been shown to be upregulated by noise (Tan et al., 2016). Additionally, local inhibition of TNF-α is equally relevant as this lowers the cochlear ability to ramp up inflammation in response to traumatic noise. Most importantly, the effectiveness of trans-tympanic ETA indicates that in an uninjured cochlea, this large molecule (∼150 kD) can cross the round window and protect against permanent NIHL. Furthermore, the *trans*-tympanic administration of ETA would avoid the global suppression of immunity associated with the systemic route.

In unanticipated NEs, we show that systemic ETA can decrease hearing loss when administered within a 2–24 h time period following NE. An interesting observation is that while post noise administration of ETA reduces the cochlear expression of TNF-α and NOX3, the high levels of TRPV1 persists and could contribute to the residual hearing loss observed in these animals. This finding could also indicate a slower turnover of TRPV1 mRNA or to differential regulation of TRPV1 and TNF-α by noise. ETA administered by the localized TT injection show a significant lowering of ABR threshold shifts when administered within 2 h of noise but not at 24 h of noise. This could reflect a slower onset of action of ETA via the TT route post injury (probably due to slower/decreased entry into the endolymph following TT injections) to prevent irreversible changes in the cochlea which are initiated within 24 h following noise. We hypothesize that systemic (peripheral) administration of the drug could enable rapid delivery to the SV via the blood stream while dampening the systemic inflammation and possibly decreasing the recruitment of immune-competent cells from systemic circulation. This would enable the cochlea to mount the resolution phase of the inflammatory cascade, which is reflected as decreased threshold shifts or amelioration of hearing loss. Thus, as a rescue treatment, the subcutaneous route provides a wider window of opportunity than the TT route.

ETA has been shown to inhibit cochlear inflammation caused by immunologic stimulation (Satoh et al., 2003; Wang et al., 2003) and cochlear electrode insertion trauma (Ihler et al., 2014). ETA has also been studied in the treatment of immune-mediated cochlea-vestibular disorders as well as autoimmune inner ear diseases in humans with varying successes (Cohen et al., 2005; Matteson et al., 2005; Van Wijk et al., 2006). Subcutaneous administration of ETA in noise-exposed guinea pigs showed hearing preservation at 8 kHz, when determined at 3 h post noise, due to the preservation of cochlear blood flow (Arpornchayanon et al., 2013). This study complements our data which indicates a window of opportunity for subcutaneous treatment from 7 days prior to NE to within 24 h post NE.

## Conclusion

This study shows that NE causes an acute phase of stress and inflammation in the cochlea, which progress to a chronic inflammatory phase that leads to damage or death of the various sensorineural cells in the cochlea. TRPV1 and TNF-α (and their subsequent synergy) are important components of this inflammatory response. Accordingly, we show that inhibition of TRPV1 or TNF-α can successfully protect and even rescue from NIHL. The translation potential of these data is significant, as it gives the physicians a window of opportunity to treat patients exposed to traumatic noise.

## Data Availability Statement

All datasets generated for this study are included in the manuscript/supplementary files.

## Ethics Statement

The animal study was reviewed and approved by Southern Illinois University School of Medicine Laboratory Animal Care and Use Committee (LACUC).

## Author Contributions

DM developed the idea for the research mentioned in this manuscript. AD, DM, and PB performed the experiments and data analysis. AD and DM wrote the manuscript and edited the figures. KK helped with gene expression analyses. VR, SS, CM, and CT critiqued and revised the manuscript.

## Conflict of Interest

The authors declare that the research was conducted in the absence of any commercial or financial relationships that could be construed as a potential conflict of interest.

## Abbreviations

ABR: auditory brainstem response
ANOVA: analysis of variance
COX-2: Cyclooxygenase-2
dB: decibel
DRG: dorsal root ganglion
EDTA: ethylenediaminetetraacetic acid
ERK1/2: extracellular signal-regulated kinase 1 and 2
ETA: Etanercept
FDA: Food and Drug Administration
GAPDH: glyceraldehyde-3 phosphate dehydrogenase
HEK: human embryonic kidney
H_2_O_2_: hydrogen peroxide
iNOS: inducible nitric oxide synthase
ICAM: intercellular adhesion molecules
IL: interleukin
JNK: c-JunN-terminal kinase
MAPK: mitogen activated protein kinase
NE: noise exposure
NGF: nerve growth factor
NIHL: noise-induced hearing loss
NOX-3: NADPH oxidase 3
OC: Organ of Corti
OHC: outer hair cell
PBS: phosphatase buffer saline
ROS: reactive oxygen species
S.E.M.: standard error of mean
SEM: scanning electron microscopy
s.c: sub-cutaneous
SGC: spiral ganglion cell
SL: spiral ligament
SPL: sound pressure level
SV: stria vascularis
TNF-α: tumor necrosis factor alpha
TRPV1: transient receptor potential vanilloid 1
TT: trans-tympanic
UB/OC-1: University Bristol/Organ of Corti-1
VR: vanilloid receptor.
